# „Crazy bitch!“ – Erlebte Aggression und Gewalt im Klinikalltag von Kinderärzten

**DOI:** 10.1007/s00112-020-01034-3

**Published:** 2020-10-16

**Authors:** Antje Dresen, Susan Lee, Holger Pfaff, Michael Weiß, Eckhard Korsch

**Affiliations:** 1 Institut für Medizinsoziologie, Versorgungsforschung und Rehabilitationswissenschaften (IMVR), Universität/Uniklinik Köln, Eupener Straße 129, 50933 Köln, Deutschland; 2 Klinik für Kinder- und Jugendmedizin, Kinderkrankenhaus Amsterdamer Straße, Amsterdamer Str. 59, 50735 Köln, Deutschland

**Keywords:** Aggressionserleben, Arzt-Patient-Angehöriger-Interaktion, Psychosoziale Belastungen, Arbeitsgesundheit, Gewaltprävention, Experience of aggression, Physician-patient-relative interaction, Psychosocial stress, Work health, Preventing violence

## Abstract

**Hintergrund:**

Es mehren sich Hinweise auf erlebte Aggressionen und Gewalt gegenüber Ärzten in Kinderkliniken. Sie werden im Kontakt mit Eltern oder Angehörigen in durch die Krankheit ihres Kindes emotional hochaufgeladenen Situationen berichtet. Mit dieser empirischen Studie wird der Frage nachgegangen, welches Ausmaß erlebtes aggressives und gewalttätiges Verhalten im Klinikalltag von Kinderärzten angenommen hat.

**Methodik:**

Ausgewertet wurden Daten von 2 bislang unveröffentlichten bundesweiten Befragungen in den Jahren 2009 (*n* = 160) und 2017 (*n* = 190). Mit dem gleichen Fragebogen wurden etwa die Formen der aggressiven Handlung wie Druckausübung, Beschimpfung, Androhung von körperlicher Gewalt, Versuch der Anwendung von Gewalt und ausgeübte Gewalt sowie die Beschreibungen der dazugehörigen Situationen abgefragt. Die Inhalte der Beschimpfung und die Art der Bedrohung konnten über offene Fragen spezifiziert werden.

**Ergebnisse:**

Etwa 4 von 5 Befragten informieren, jemals Ziel einer aggressiven Handlung von Eltern oder Angehörigen gewesen zu sein. In der deskriptiven Gegenüberstellung der Erhebungszeiträume betrachten die Befragten die Problematik des aggressiven Verhaltens als zunehmend relevant für ihren beruflichen Alltag. Im Jahr 2017 betonten dies 3 von 4 Befragten (71,0 %) gegenüber nur jedem Zweiten (51,9 %) im Jahr 2009. Vereinzelt wird von bis zu 60 Situationen berichtet, in beiden Befragungswellen im Median 4,0-mal/Jahr.

**Diskussion:**

Erlebte Aggressionen und Gewalt gehören vielfach und vermehrt zum Klinikalltag auf den pädiatrischen Stationen – angefangen von Beleidigungen bis hin zu ausgeübter körperlicher Gewalt. Präventionsstrategien wie vorbeugende Trainings zu Kommunikation und Deeskalation werden explizit gewünscht.

## Abstract (en)

**Background:**

The indications for experienced aggression and violence towards doctors in children’s hospitals are increasing and are the focus of this study. They are reported in contact with parents or relatives in emotionally highly charged situations caused by their child’s illness. This empirical study investigated the extent to which experienced aggressive and violent behavior has been received by pediatricians in their everyday work in hospitals.

**Methods:**

Data from two previously unpublished nationwide surveys in 2009 (*n* = 160) and 2017 (*n* = 190) were analyzed. Using the same questionnaire, the forms of aggressive action, such as exerting pressure, insulting, threatening physical violence, attempting to use violence and actually using violence as well as the descriptions of the associated situations were questioned. The wording of the insults and the type of threat could be specified via open questions.

**Results:**

Approximately four out of five respondents said they have been the target of an aggressive action by parents or relatives. In 2017 approximately 3 out of 4 respondents (71.0%) considered the problem of aggressive behavior to be relevant to their everyday work compared to only every second respondent (51.9%) in 2009. Individual respondents reported up to 60 situations, in both survey waves at a median of 4.0 times per year.

**Conclusion:**

Experienced aggression and violence are often and increasingly part of everyday clinical life in the pediatric wards, ranging from insults to physical violence. Prevention strategies, such as preventive training for communication and de-escalation are explicitly desired.

## Hintergrund

Aggressionen und gewalttätige Handlungen gegenüber Ärzten stehen immer mehr im Fokus. Unter Hausärzten werden bis zu 90 % im Laufe ihres Berufslebens mit aggressivem Verhalten von Patienten konfrontiert – von Beschimpfungen und Beleidigungen bis hin zu Angriffen mit Gegenständen und Waffen [13]. Bis zu 25 % der Niedergelassenen geben überdies an, bereits Erfahrungen mit körperlicher Gewalt in der Praxis gemacht zu haben [12].

Gegenüber Ärzten in Kinderkliniken wird das Phänomen der Aggressionen und Gewalt im öffentlich-medialen und wissenschaftlichen Diskurs nur randständig wahrgenommen. Dabei mehren sich gerade dort Hinweise im Kontakt mit Eltern oder Angehörigen in durch die Krankheit ihres Kindes emotional hochaufgeladenen Situationen.

Lediglich Mackin [8] für Irland und daran anlehnend für den bundesdeutschen Raum Korsch et al. [5] haben bislang einen systematischen Einblick in die erlebten Aggressionen aus Sicht der Kinderärzte gegeben. Danach waren bereits 9 von 10 (89,9 %, *n* = 228), in einer Umfrage von Kölfen [4] sogar 99 % der Befragten schon einmal Ziel eines aggressiven Vorfalls gewesen.

Vor diesem Hintergrund wird mit diesem Beitrag abermals der Frage nachgegangen, welches Ausmaß aggressives und gewalttätiges Verhalten von Angehörigen im Klinikalltag von Kinderärzten angenommen hat. Ausgewertet wurden dafür Daten von 2 bislang unveröffentlichten bundesweiten Befragungen in den Jahren 2009 und 2017, die beide auf dem gleichen Fragebogen von Korsch et al. [5] basieren.

## Methodik

Diese Studie fußt auf 2 voneinander unabhängigen Befragungswellen von in Kliniken tätigen Kinderärzten in Deutschland. Auf jeweils eine einmalige bundesweite Aussendung von 387 (2009) bzw. 407 Fragebogen (2017) an die jeweiligen Assistentenvertreter aller pädiatrischen Abteilungen und Kinderkliniken haben sich im Jahr 2009 *n* = 160 und im Jahr 2017 *n* = 190 Ärzte zurückgemeldet.

Im Fragebogen werden die Formen der aggressiven Handlung wie Druckausübung, Beschimpfung, Androhung von körperlicher Gewalt, Versuch der Anwendung von Gewalt und ausgeübte Gewalt sowie die Beschreibungen der dazugehörigen Situationen abgefragt. Zugleich konnten die Befragten etwa die Inhalte der Beschimpfung und die Art der Bedrohung über offene Fragen spezifizieren. Weiterhin wurde erhoben, ob die aggressive Handlung eine für sie persönliche Belastung war und, wenn ja, in welcher Form. Darüber hinaus interessierte, ob die aggressive Handlung die Art der Ausübung der kinderärztlichen Tätigkeit (auch nur kurzfristig) beeinflusste. Dazu wurden weitere Parameter wie Geschlechtszugehörigkeit, Dauer der Tätigkeit in der Pädiatrie, berufliche Position der Befragten, Bettenanzahl in der Kinderklinik, Postleitzahl des Ortes der Kinderklinik und Einwohnerzahl des Ortes exploriert.

Die Auswertung erfolgte primär mittels univariater Analysen und Prüfungen bivariater Zusammenhänge. Gruppenverteilungen werden in Prozent angegeben. Bei nichtnormalverteilten Werten ist der Median berechnet worden. Zusammenhänge zwischen 2 nominal- bis ordinalskalierten Merkmalen sind mittels eines Pearson-Chi-Quadrat-Tests geprüft worden. Unterschiede zwischen nichtnormalverteilten ordinalen Daten sind mit dem Mann-Whitney-U-Test als nichtparametrischer Test und Unterschiede zwischen zentralen Tendenzen (mehr als 2 Stufen) mehrerer unabhängiger Stichproben mit dem Kruskal-Wallis-Test berechnet worden. Als Irrtumswahrscheinlichkeit wurde ein *p*-Wert ≤0,05 als schwach signifikant, *p* ≤ 0,01 als signifikant und *p* ≤ 0,001 als sehr/hochsignifikant definiert [6, 7]. Die Berechnung erfolgte mit dem Programm SPSS 25 (International Business Machines Corporation, New York, USA) für Windows (Microsoft, Redmond, Washington, USA). Dabei ist im Textfluss nur dann der *p*-Wert angegeben, wenn er auf Signifikanzen verweist. Die Antworten auf die offenen Fragen sind kategoriengeleitet inhaltsanalytisch ausgewertet und sodann über Häufigkeitsverteilungen subsumiert worden. Dies geschah unter Zuhilfenahme des Programms MAXQDA 2018 (Verbi GmbH, Berlin, Deutschland).

## Ergebnisse

### Zusammensetzung der Stichprobe

Von den 160 befragten Ärzten im Jahr 2009 befanden sich die meisten (40,5 %) in der Position eines Assistenzarztes in der fortgeschrittenen Ausbildung. Dies trifft auch auf die 190 befragten Ärzte im Jahr 2017 zu (48,4 %). Gut ein Viertel ist in beiden Befragungsjahren Facharzt (25,9 % bzw. 26,6 %), gefolgt von Oberarzt (17,1 % bzw. 9,0 %), Assistenzarzt in den ersten 18 Monaten (früher Arzt im Praktikum) mit 8,2 % bzw. 13,3 % sowie Chefarzt mit 8,2 % bzw. 2,7 %.

Knapp die Hälfte (51,3 %) der Befragten im Jahr 2009 ist weiblich, 48,7 % männlich. Dahingegen sind zwei Drittel im Jahr 2017 weiblich (65,8 % zu 32,1 %). Die Mediziner sind im Median seit 6,0 Jahren (2009) bzw. seit 5,0 Jahren (2017) in der Pädiatrie beschäftigt. Die meisten arbeiten in Kliniken, deren Bettenanzahl im Median 54 (Jahr 2009) bzw. 50 Betten (Jahr 2017) betrug und die in Orten zwischen durchschnittlich 90.000 (Median im Jahr 2009) bzw. 113.500 (Median im Jahr 2017) Einwohnern angesiedelt waren.

### Formen und Ausprägungen der aggressiven und gewaltsamen Handlungen

In beiden Befragungen informieren etwa 4 von 5 Befragten, jemals Ziel einer aggressiven Handlung von Eltern oder Angehörigen gewesen zu sein. Dabei ist der Anteil jener, die von solchen Erfahrungen berichten, vom Jahr 2009 (78,8 %) zu 2017 (87,9 %) größer geworden. Im Vergleich der beiden Erhebungszeiträume betrachten die Befragten die Problematik des aggressiven Verhaltens als zunehmend relevant für ihren beruflichen Alltag. Im Jahr 2017 betonten dies 3 von 4 Befragten (71,0 %) gegenüber nur jedem Zweiten (51,9 %) im Jahr 2009. Vereinzelt wird von bis zu 60 Situationen berichtet, in beiden Befragungswellen im Median 4,0-mal/Jahr. Dabei macht es im Jahr 2017 einen signifikanten Unterschied, welche berufliche Position der Befragte in Bezug auf die Anzahl der erlebten aggressiven Handlungen insgesamt hat (*p* = 0,008). Je niedriger die Position des Antwortenden in der Klinik, desto häufiger wird von Aggressionen berichtet. Dazu korrelieren weder die Geschlechtszugehörigkeit noch die Größe der Stadt in beiden Jahren mit den Angaben zu den in der Häufigkeit erlebten Aggressionen.

Die Formen der aggressiven Handlungen beziehen sich u. a. auf *Druckausübung durch Androhung*. Dies betrifft etwa die diagnostische oder therapeutische Entscheidung durch Androhung einer Beschwerde bei Vorgesetzen (48,1 % im Jahr 2009, 51,1 % im Jahr 2017), bei der Verwaltung des Krankenhauses (38,1 % zu 41,1 %), Meldung an die Medien (21,3 % zu 18,9 %) und Einschaltung eines Rechtsanwaltes (20,6 % zu 27,9 %). Während die Prävalenzen in dieser Kategorie über beide Befragungsjahre relativ stabil geblieben sind, ist das vergleichsweise größere Spektrum der genannten Inhalte im Jahr 2017 qualitativ auffällig. Es reicht von „*Meldung an die Ärztekammer*“ oder an die „*Polizei*“ bis zu „*Es wurde ein Handy-Foto von mir gemacht.*“, „*Anklage und Entziehung der Approbation*“, „*im Internet diskreditierende Patientenportalbeurteilung*“, „*Ermordung*“, „*Bombendrohung*“ bzw. „*Bombendrohung gegen Klinik aufgrund einer Wartezeit von 1* *h in der Ambulanz*“, „*Wenn das Kind stirbt, bringe ich Dich um*“ und „*Ich mache Sie fertig. Sie werden Ihres Lebens nicht mehr froh*“. Eine Befragte berichtet: „*Vater eines Kindes tritt fälschlicherweise als Redakteur der Bildzeitung auf*“. Dagegen werden im Jahr 2009 zur erlebten Druckausübung durch Androhung lediglich „*Ärztekammer*“ und „*körperliche Aggression*“ angegeben.

**Figure 1:**
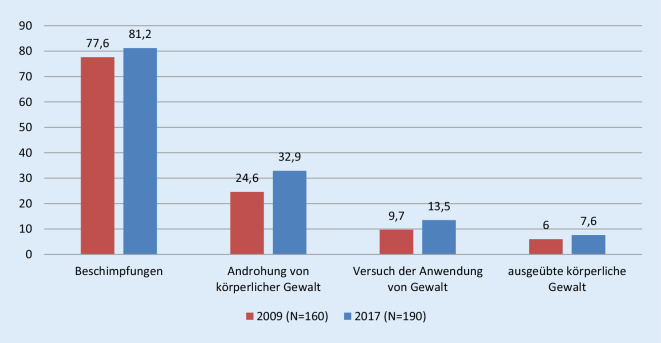

Bei den erlebten Formen von Aggressionen dominieren weiterhin *Beschimpfungen*, gefolgt von *Androhung körperlicher Gewalt, Versuch der Anwendung von Gewalt* und *ausgeübte körperliche Gewalt*. Damit hat sich das berichtete Problem im Vergleich der beiden Befragungsjahre auf allen Ebenen verstärkt (Abb. 1).

Die erlebten Beschimpfungen und Androhungen von körperlicher Gewalt sowie der Versuch der Anwendung von Gewalt zeigen sich geschlechtsunabhängig. Die ausgeübte körperliche Gewalt passierte den männlichen Befragten nach deren Angaben im Jahr 2017 jedoch häufiger (*p* ≤ 0,001), wohingegen für das Jahr 2009 lediglich eine Tendenz Richtung männlicher Betroffener bestand (*p* ≤ 0,18).

**Figure 2:**
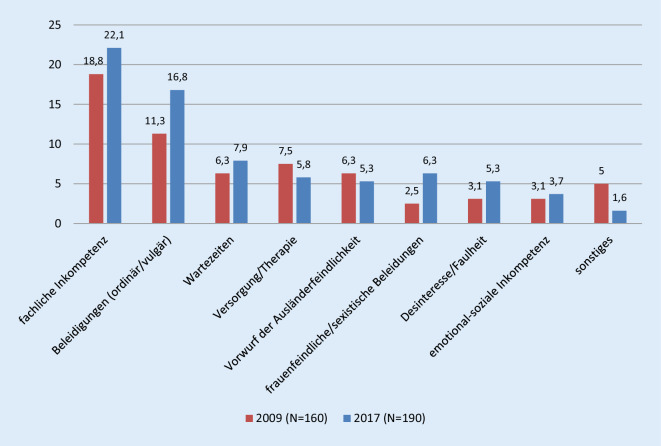

Aus den Beschimpfungen lassen sich auf der inhaltlichen Ebene die Kategorien fachliche Inkompetenz, ordinäre/vulgäre Beleidigungen, Wartezeiten, Versorgung/Therapie, Vorwurf der Ausländerfeindlichkeit, frauenfeindliche und sexistische Beleidigungen, Desinteresse/Faulheit, emotional-soziale Inkompetenz und Sonstiges rekonstruieren (Abb. 2). In beiden Befragungsjahren überwiegt der Vorwurf der fachlichen Inkompetenz, gefolgt von Beleidigungen. Dabei haben diese beiden Ausprägungen von Beschimpfungen überproportional zugenommen. Bemerkenswert ist dazu eine fast Verdreifachung der frauenfeindlichen und sexistischen Beleidigungen. In der dazugehörigen Tab. 1 sind die Inhalte der Beschimpfungen als wortwörtliche Ankerbeispiele aufgeführt.

**Table 1:** 

Inhalte der Beschimpfungen	Angaben/Beispiele (wortwörtlich)
Fachliche Inkompetenz	*Ich sei dumm und unfähig, inkompetenter Haufen*
Beleidigungen (ordinär/vulgär)	*Penner, Arschloch, Wichser, Scharlatan*
Wartezeiten	*Unprofessionelle Arbeit wegen langer Wartezeit*
Versorgung/Therapie	*Schlechte Orga, Essen, Missachtung der Hygieneregeln*
Vorwurf der Ausländerfeindlichkeit	*Hitler, Nazi, Frau Doktor Hitler, Rassist, Ausländerfeind, Judenarzt, nur von einem Ausländer behandelt*
Frauenfeindliche/sexistische Beleidigungen	*Crazy bitch, Schlampe, Hure, schwule Sau, blöde Fotze, Schwuchtel, blöde Kuh, Ziege, Gebärmaschine, Flittchen*
Desinteresses/Faulheit	*Ignorante Ärzte, unzureichende Fürsorge, mangelndes Engagement*
Emotional-soziale Inkompetenz	*Keine Gefühle, fehlende Empathie, fehlende Sensibilität*
Sonstiges	*Menschenunwürdig, steht morgen in der BILD, wollen nur Geld verdienen, dein Auto mache ich mit meinem Bagger kaputt, ich mache Sie fertig, Sie werden Ihres Lebens nicht mehr froh*

**Figure 3:**
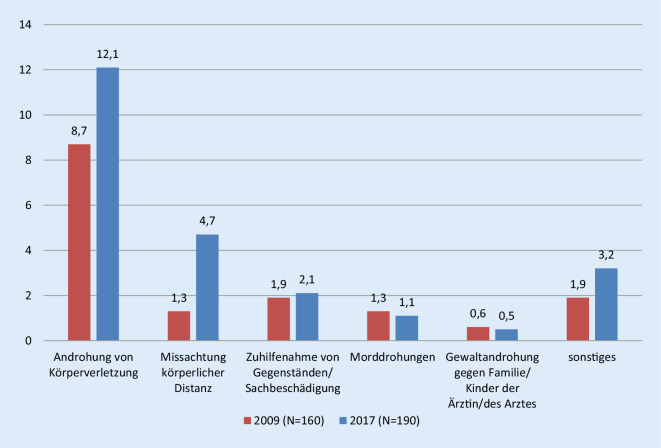

In der Kategorie „Inhalte angedrohter Gewalt“ fallen ins Gewicht: Androhung von Körperverletzung, Missachtung körperlicher Distanz, Zuhilfenahme von Gegenständen/Sachbeschädigung, Morddrohungen und Gewaltandrohung gegen die Familie/Kinder der Ärztin/des Arztes (Abb. 3). Auffällig ist der Unterschied der erlebten Androhung von Körperverletzung und Missachtung körperlicher Distanz zwischen den Jahren 2009 und 2017. In Tab. 2 sind die Inhalte der Beschimpfungen wortwörtlich aufgeführt.

**Table 2:** 

Inhalte angedrohter Gewalt	Angaben/Beispiele (wortwörtlich)
Androhung von Körperverletzung	*Drohung zu beißen bei HIV-positivem Vater, würgen, Prügel, ich mache Dich fertig, ich schneide Euch die Ohren ab*
Missachtung körperlicher Distanz	*Vater baut sich mit rotem Kopf vor mir auf, Auflauern im Parkhaus, am Kittel festgehalten, Aufstellung dreier Männer 1,90* *m vor mir, langsames Auf-mich-Zugehen in Dreierreihe*
Zuhilfenahme von Gegenständen/Sachbeschädigung	*Bedrohung mit Schlüsselbund, Elektroschocker, Axt, fackel’ das ganze KH nieder*
Morddrohungen	*Ich stech’ dich ab, ich bringe Dich um, ich bringe Euch alle um*
Gewaltandrohung gegen Familie/Kinder der Ärztin/des Arztes	*Bedrohung meiner Familie*
Sonstiges	*Unter Alkoholeinfluss, Drogen, ich werde Sie finden, können etwas erleben, wenn meinem Kind etwas passiert, sind Sie dran*

**Figure 4:**
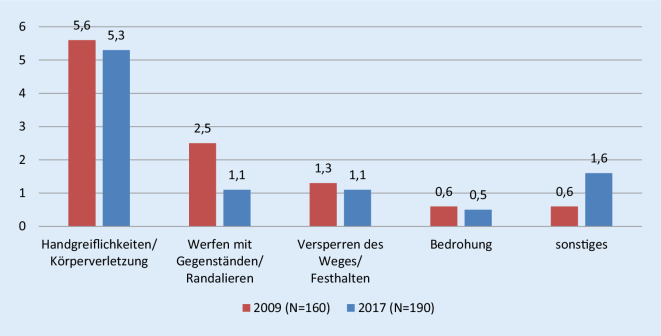

Unter „Inhalte versuchter Gewaltanwendung“ sind schließlich Handgreiflichkeiten/Körperverletzung, Werfen mit Gegenständen/Randalieren, Versperren des Weges/Festhalten, Bedrohung und Sonstiges rekonstruiert worden (Abb. 4; Tab. 3).

**Table 3:** 

Inhalte versuchter Gewaltanwendung	Angaben/Beispiele (wortwörtlich)
Handgreiflichkeiten/Körperverletzung	*Am Arm gepackt, Faustschläge, kneifen, schlagen, wegschubsen*
Werfen mit Gegenständen/Randalieren	*Bewerfen, Stuhl herumwerfen, Türen eintreten, Scheibenbruch*
Versperren des Weges, Festhalten	*Am Kittel festgehalten, in die Enge gedrängt*
Bedrohung	*Bedrohung mit Elektroschocker*
Sonstiges	*Waffe auf Untersuchungsliege gelegt, hämmern an die Untersuchungszimmertür*

Auffällig ist, dass Handgreiflichkeiten/Körperverletzung und Werfen mit Gegenständen/Randalieren in beiden Befragungsjahren als Nennungen dominierten. Darüber hinaus wird gerade hier deutlich, dass die erlebten Aggressionen unterschiedlich stark empfunden und entsprechend zugeordnet werden. So wird das „am Kittel festhalten“ nicht nur als angedrohte Gewalt (Tab. 2), sondern auch versuchte Gewaltanwendung (Tab. 3) oder gar als ausgeübte Gewalt erlebt (Tab. 4). Das Überschreiten der als angemessen empfundenen körperlichen Distanz empfinden einige als Androhung von Gewalt (Tab. 2), andere ordnen diese unter ausgeübter Gewalt ein (Tab. 4).

**Table 4:** 

Inhalte ausgeübter Gewalt	Angaben/Beispiele (wortwörtlich)
Schläge, Tritte	*Faustschlag, Tritt gegen den Kopf*
Körperverletzungen wie Würgen, Kratzen, Kneifen, Beißen, Schütteln etc.	*Greifen am Kittelkragen und schütteln, handgreifliche Auseinandersetzung*
Gewaltsame Bedrohung	*Messer an die Kehle gehalten*
(Weg‑)Stoßen	*Anrempeln, wegstoßen*
Sonstiges	*Telefonterror, zu nahe gekommen, Infusionsspender von der Wand gerissen, schreien*

**Figure 5:**
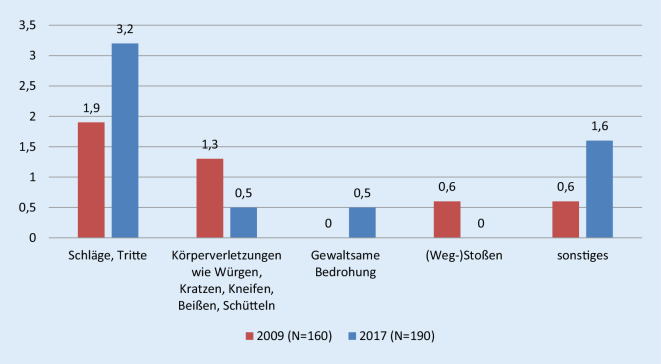

Die Inhalte ausgeübter Gewalt (Abb. 5; Tab. 4) implizieren letztlich v. a. Schläge und Tritte sowie weitere Körperverletzungen wie Würgen, Kratzen, Kneifen, Beißen, Schütteln. Hinzu kommen gewaltsame Bedrohung, (Weg‑)Stoßen und Sonstiges. Dabei haben sich die erlebten Formen der schwereren Körperverletzung wie Schläge und Tritte fast verdoppelt.

Mit Blick auf die Verschriftlichungen der dazugehörigen Situationen werden hochemotionale Szenen deutlich. Ein Befragter berichtet nach dem Tod eines Kindes: „*Die Mutter würgte mich, vier Männer mussten sie von mir abhalten*.“ Ein anderer schildert eine Situation in der Notfallambulanz: *„Kind war nicht wirklich krank. Musste zur Reanimation zu einem anderen Kind. Danach war der Vater des nichtkranken Kindes stark erregt, wollte unbedingt Medikamente haben. Als ich die verweigert hatte, bedrohte er meine Pflegekraft. Als ich dazwischen bin, wurde ich ins Gesicht geschlagen“*. Weiterhin wird mehrfach von stark alkoholisierten Vätern und von schwierigen familiären Verhältnissen gesprochen.

### Persönliche Belastungen und Beeinflussung der kinderärztlichen Tätigkeit

**Figure 6:**
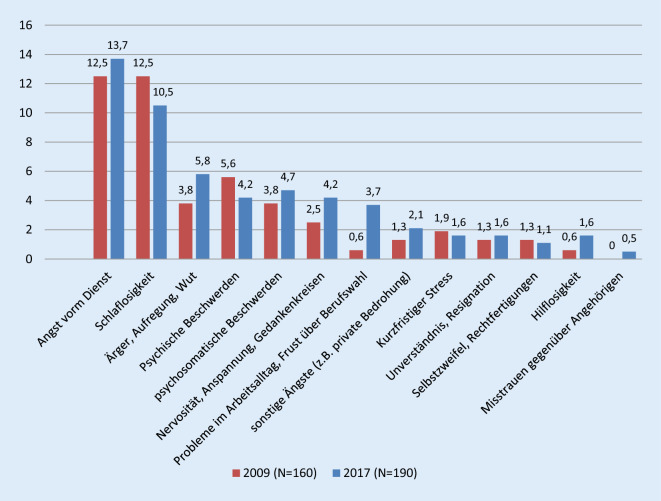

Knapp die Hälfte aller Befragten (47,8 % im Jahr 2009, 47,6 % im Jahr 2017) gibt an, die Aggressionen als persönlich belastend empfunden zu haben. Dabei haben sich keine signifikanten Unterschiede zwischen den Geschlechtern gezeigt. Über beide Befragungsjahre betrachtet hatten die meisten Angst vorm Dienst, berichten über Schlaflosigkeit, Ärger, Aufregung, Wut sowie psychische und psychosomatische Beschwerden (Abb. 6). Im Besonderen Angst vorm Dienst hatten jedoch häufiger Frauen (*p* ≤ 0,05 in 2017). Die berufliche Position des Antwortenden bleibt von den berichteten Belastungen statistisch unberührt.

Über den Indexfall hinaus berichtet ungefähr ein Drittel der jeweils Befragten (35,3 % im Jahr 2009, 35,5 % im Jahr 2017) über Folgen für die Art der Ausübung der kinderärztlichen Tätigkeit. Dabei wird hauptsächlich der Umgang mit den Eltern und Angehörigen (25,6 % für 2009 bzw. 27,9 % für 2017) angeführt, gefolgt von der Beeinflussung diagnostischer oder therapeutischer Entscheidungen (7,5 % für 2009 bzw. 7,4 % für 2017) und dem Umgang mit den Mitarbeitern (5,8 % für 2009 bzw. 7,5 % für 2017).

In beiden Befragungsjahren nahmen nur jeweils 4 Befragte professionelle Hilfe nach den Vorfällen in Anspruch. Die Kontaktpersonen im Jahr 2009 waren der ärztliche Direktor, ein Krankenpfleger und ein Psychotherapeut. Im Jahr 2017 wurden der psychosoziale Dienst und Kollegen per Supervision im Team informiert. Jeweils einer rief die Polizei.

Eine Meldung erging hingegen in knapp der Hälfte aller Fälle (49,6 % in 2009, 56,4 % in 2017). Dabei rangiert der Vorgesetzte mit 36,5 % (2009) bzw. 45,3 % (2017) vor der Polizei (9,4 % in 2009 bzw. 9,5 % in 2017) und der Verwaltung (4,4 % in 2009 bzw. 8,9 % in 2017).

Eine Präventionsmaßnahme als Training im Umgang mit solchen Situationen erachten 84,4 % im Jahr 2009 und 93,0 % in 2017 für sinnvoll. Allerdings hatte nur etwa jeder Fünfte die Möglichkeit, daran teilzunehmen (18,3 % in 2009 bzw. 20,7 % in 2017).

## Diskussion

Neben dem Rettungswesen zeugt gerade der pädiatrische Bereich von emotional hochaufgeladenen Situationen, in denen sich oftmals Aggressionen und Gewalt entladen können. Mit Blick auf die letzten knapp 2 Jahrzehnte scheint sich das Problem in zahlreichen Facetten verstärkt zu haben. So geht aus Korsch et al. [5] noch hervor, dass die Häufigkeit eines solchen Vorfalls im Median einmal pro Jahr betrug. Im Rahmen dieser erneuten Befragungen hat sich die Anzahl der berichteten Vorfälle in den Jahren 2009 und 2017 auf 4 im Jahr (4,0 Median) vervierfacht.

Wie bei Kölfen [4] ist perspektivisch auffällig, dass es einen signifikanten Unterschied macht, welche berufliche Position der Befragte in Bezug auf die Anzahl der erlebten aggressiven Handlungen insgesamt hat. Je niedriger die Position des Antwortenden in der Klinik, desto häufiger wird von Aggressionen berichtet. Die Hemmschwelle, aggressiv zu werden, scheint einem Assistenzarzt gegenüber kleiner zu sein als gegenüber einem hierarchisch höher gestellten Fach‑, Ober- und Chefarzt mit entsprechend mehr zugewiesener Expertise. Mit Blick auf die Inhalte der Beschimpfungen beziehen sich so auch die meisten Anfeindungen auf den Vorwurf der fachlichen Inkompetenz. Eine weitere Deutung zu den unterschiedlichen Angaben ist, dass die erfahrenen Ärzte mit den Dienstjahren mehr Distanz zu solchen Ereignissen eingenommen und sie in einem gewissen Rahmen „normalisiert“ haben.

Darüber hinaus ist frappant, dass alle Formen erlebter Aggressionen im Jahresvergleich 2009 und 2017 quantitativ zugenommen haben und qualitativ bedeutungsschwer aufgeladen sind. Auf Basis von Korsch et al. [5] haben etwa noch 74,5 % aller Befragten Beschimpfungen erlebt, im Jahr 2009 waren dies bereits 77,6 % und 2017 81,2 %. Dabei sind gerade die frauenfeindlichen/sexistischen Beleidigungen angestiegen, was wiederum sowohl mit Bezug zur Häufigkeit der Vorkommnisse als auch im Hinblick auf die vermehrte Sensibilität gegenüber diesen Formen der erlebten Aggression interpretiert werden kann.

Innerhalb dieser Studie lassen sich die konkreten Gründe für die erlebten Übergriffe mitunter aus den verschriftlichten Inhalten zu den verbalen Aggressionen rekonstruieren. Diese beziehen sich bei den erlebten Beschimpfungen v. a. auf Wartezeiten, Unzufriedenheiten mit der Versorgung und Therapie, empfundenes Desinteresse am Kind und mangelndes Einfühlungsvermögen. Internationale Studien stützen diese Erkenntnisse [1, 9, 11]. Auch die Ärztekammern sehen als Hauptauslöser für Aggression und Gewalt lange Wartezeiten, die Frage, in welcher Reihenfolge Patienten behandelt werden dürfen, und fehlendes Verständnis dafür, dass Notfälle Vorrang haben. Dazu geht es um die Sorge um Angehörige, vermutete und tatsächliche Fehlbehandlungen, Zuzahlungen, interkulturelle Missverständnisse und falsche Versprechungen [3].

Von allen befragten Ärzten hat fast die Hälfte angegeben, die Aggressionen als persönlich belastend empfunden zu haben. Sie äußerten v. a. Angst vorm Dienst, Schlaflosigkeit, Ärger, Aufregung, Wut sowie psychische und psychosomatische Beschwerden. Dies sind Auswirkungen, die bereits Hobbs [2] in Bezug auf Klinikärzte mit Aggressionserfahrungen feststellte. Mehr als ein Drittel aller Befragten in dieser Studie hat überdies berichtet, dass die Art der Ausübung der kinderärztlichen Tätigkeit durch das Erlebnis einer aggressiven Handlung beeinflusst worden sei. Dieses Ergebnis fügt sich in die internationale Studienlage, derzufolge zwischen 26,5 und 37,5 % der Befragten verdeutlichen, die Beziehung zu den Patienten und deren Angehörigen habe sich negativ verändert [1, 10].

Im vorliegenden Beitrag richtet ein Drittel der Befragten seine berufliche Tätigkeit nach den problematischen Indexfällen neu aus – im Umgang mit Eltern, Angehörigen und Mitarbeitern sowie bei diagnostischen und therapeutischen Entscheidungen. Diese Anpassungen des ärztlichen Handelns bedürfen zukünftig einer differenzierteren Betrachtung. Sie geht einher mit Fragen des Umgangs mit solchen Situationen und etwaigen Präventionsstrategien wie vorbeugende Trainings zu Kommunikation und Deeskalation, die sich die meisten Befragten wünschen.

### Limitationen

- Obwohl die Ergebnisse beider Befragungszeitpunkte gegenübergestellt und im Verhältnis zueinander interpretiert werden, stehen sie in keinem statistischen Zusammenhang. Zwar wurde in den Jahren 2009 und 2017 exakt der gleiche Fragebogen an alle Assistentenvertreter aller pädiatrischen Abteilungen und Kinderkliniken versendet. Auch bestehen durchaus Ähnlichkeiten zwischen den Kollektiven mit Blick auf die prozentuale Verteilung der beruflichen Positionsinhaber. Die Ergebnisse basieren jedoch nicht auf einer Panelstudie. Es ist von einer Personalfluktuation über die Jahre auszugehen.
- Die Problemdeutungen beziehen sich nicht ausschließlich auf die (wenigen) statistisch-signifikanten Auffälligkeiten. Die kategoriengeleiteten Antworthäufigkeiten und Auffälligkeiten bei verschiedenen Merkmalsausprägungen sind darüber hinaus deskriptiv – also v. a. statistisch beschreibend und ordnend – in die Analyse eingeflossen.
- Die dargelegte Problemlage der erlebten Aggressionen und Gewalt kann an der erhöhten Anzahl der Vorkommnisse oder an einer sensibleren, auch medial forcierten Wahrnehmung der Thematik im beruflichen Alltag liegen.

## Ausblick

Um gesunderhaltende Arbeitsbedingungen der Ärzteschaft und eine bedürfnisorientierte Versorgung erkrankter Kinder nachhaltig zu gewährleisten, gilt es, sich v. a. der kommunikativen Herausforderungen im Geflecht Arzt-Patient-Angehöriger anzunehmen. Kernelemente dafür können sein: Vertrauensaufbau, Empathie, Transparenz, Aufklärung (auch über organisatorische Abläufe und Wartezeiten), Interkulturalität, Deeskalation, Selbstfürsorge und Supervision. Darüber hinaus wird von Interesse sein nachzuverfolgen, ob die derzeitige positive Stimmung gegenüber Helfern im Rahmen der SARS-CoV-2-Pandemie auf erlebte Aggression und Gewalt im Klinikalltag von Kinderärzten Auswirkungen haben wird.

## Fazit für die Praxis

- Erlebte Aggressionen und Gewalt gehören vielfach und vermehrt zum Klinikalltag auf den pädiatrischen Stationen – angefangen von Beleidigungen bis hin zu ausgeübter körperlicher Gewalt.
- Die Aggressionserfahrungen wirken für viele Klinikärzte psychisch, psychosomatisch und auf ihr ärztliches Handeln nach.
- Kommunikationstrainings im Geflecht Arzt-Patient-Angehöriger und Möglichkeiten der Deeskalation werden gewünscht. Eine institutionelle Verankerung jener Maßnahmen kann sich präventiv auf die Gesunderhaltung der Ärzteschaft und die bedürfnisorientierte Behandlung der Kinder auswirken.
